# An Infertile Man with Complementary Isochromosome of 46, XY, I(5)(P10),I(5)(Q10): A Case Report

**Published:** 2019-09

**Authors:** Yu HE, Bo LIU, Hui-Ling WU, Shan HUANG, Yun-Wei QI, Hui-Han ZHAO, Xue QIN, Shan LI

**Affiliations:** 1. Department of Clinical Laboratory, The First Affiliated Hospital of Guangxi Medical University, Nanning, P.R. China; 2. Department of Nursing, The First Affiliated Hospital of Guangxi Medical University, Nanning, P.R. China

**Keywords:** Isochromosome, Infertility, Secondary hypothyroidism

## Abstract

Chromosome aberration is associated with infertility and isochromosome is a rare chromosomal abnormality potentially associated with infertility or multiple miscarriages. Here we report a man of 29-yr-old diagnosed of infertility from China with rare isochromosome. Peripheral blood lymphocytes were obtained for chromosome G banding karyotyping. Hormones and other tests were performed. The semen analysis revealed Azoospermia. Complementary isochromosome of 46, XY, i(5)(p10),i(5)(q10) was identified. This is the first report of Complementary isochromosome of 46, XY, i(5)(p10),i(5)(q10).

## Introduction

Azoospermia was detected in approximately 15% to 20% of infertile men, and the most common chromosomal alterations as Robertsonian translocations and sex chromosome aberrations are responsible for about 8.0% of all azoospermia cases (1) Complementary isochromosome is a group of rare karyotype. A few of them have been reported. An infertile oligo asthenoteratozoospermic man has a karyotype 46,XY, i(9)(p10),i(9)(q10) (2). A boy with growth retardation had a karyotype 46,XY,i(7)(p10),i(7)(q10) (3). A healthy male had a karyotype 46,XY, i(2)(p10),i(2)(q10) (4).

In this report, we present an unusual karyotype of 46,XY,i(5)(p10),i(5)(q10) in an azoospermia man.

## Case Report

An infertile man of 29-yr-old presented to our laboratory for karyotype analysis after 3 years of sexual intercourse without conception. His wife was 28-yr-old, did not have any fertility problems. No history of infertility was noted in his family. The patient was suffered from hyperthyroidism years ago.

Informed consent was obtained from the patient and this study was approved by the Ethics Committee of the First Affiliated Hospital of Guangxi Medical University.

After iodine 131 treatment, the disease turned into hypothyroidism now. The patient underwent physical examination, semen analysis, hormonal exploration, antisperm antibody test, B-mode ultrasonography and genetic investigations. Physical examination revealed normal testes with first degree varicocele in left side, B-mode ultrasonography revealed that there were several spherical or tubular echo free zone between left scrotum and inguinal, and it could be flattened when forcing the probe unit. The maximum internal diameter of the tube was 0.20 cm, and it expanded to 0.24 cm in Valsalva test. No evidence of gynaecomastia and secondary sexual characteristics were normal.

Laboratory investigation revealed azoospermia in semen analysis (performed three times) associated with high levels of serum estradiol (112.00 pg/ml); follicle stimulating hormone (FSH), luteinic hormone (LH), prolactin (PRL), progesterone, testosterone were within the normal range (8.22 mUI/ml, 4.23 mUI/ml, 9.01 ng/ml, 0.55 ng/ml, 2.81 ng/ml). Antisperm antibody in serum and Mixed Agglutination Reaction (MAR) in semen were both negative. No extended or partial Y chromosome microdeletions were found.

**Fig. 1:**
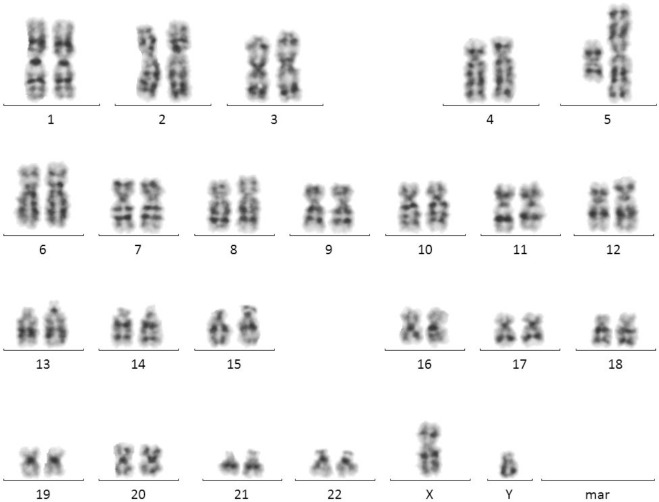
Karyotype of complementary isochromosome 5: 46,XY,i(5)(p10),i(5)(q10)

After receiving informed consent, cytogenetic investigation was performed on lymphocyte chromosomes obtained from peripheral blood. Metaphases were studied with a standard G banding procedure. The karyotype revealed the presence of a complementary isochromosome of 46, XY, i(5)(p10), i(5)(q10) (Fig. 1).

## Discussion

As far as known, this is the first report of a patient carrying isochromosome 5 associated with male infertility. In our case, karyotype analysis by using G banding at the 550 band level revealed no obvious deletion or loss of chromosomal.

Uniparental disomy (UPD) is defined as the inheritance of both homologues of a pair of chromosomes from one parent only. Its formation mechanisms associated with an isochromosome could be explained in several pathogenetic ways. The most common theory is that there is a mistake in centromeres during meiosis, which produces a gamete containing isochromosomes, which is fertilized with a normal gamete, and occurs homologous chromosome loss during subsequent mitosis (5,6).

After a large cohort research, Simmonds MJ confirmed that hyperthyroidism is related with chromosome 5q31-33 (7). After treatment of I131, the patient developed hypothyroidism. Ceccarelli C believed that the damage caused by hyperthyroidism itself may be higher than that caused by the treatment of iodine 131 (8). Nikoobakht MR found that hypothyroidism affects the erectile function, reduces the number of sperm, increases the rate of sperm malformation and reduces the sperm motility (9).

Bian X found that male hyperthyroidism and hypothyroidism group E2 was significantly higher than that in the control group. As a result of the changes in thyroid hormone levels, the hypothalamic pituitary gonadotropin axis dysfunction occurred (10). Varicoceles are the most common cause of infertility in men, although not all males with varicocele experience infertility. In fact, most men with varicocele have normal spermatogenesis. Our patient suffered from first degree varicocele which might not be the decisive factor of azoospermia.

This is a case of azoospermia with multiple symptoms and of complex pathogenesis. To explore eventual implication of one or more of these apoptosis regulator genes in spermatogenesis impairing observed in our case, further analysis by FISH using specific probes is needed; Clinicians should first analyze chromosome karyotype before providing assisted reproductive technology, so as to exclude rare chromosomal abnormalities, and ensure a good prognosis of assisted reproduction.
